# Oversized vein grafts develop advanced atherosclerosis in hypercholesterolemic minipigs

**DOI:** 10.1186/1471-2261-12-24

**Published:** 2012-03-31

**Authors:** Troels Thim, Mette Kallestrup Hagensen, Arne Hørlyck, Ludovic Drouet, William P Paaske, Hans Erik Bøtker, Erling Falk

**Affiliations:** 1 Atherosclerosis Research Unit, Department of Cardiology, Aarhus University Hospital and Institute of Clinical Medicine, Aarhus University, Aarhus, Denmark; 2 Department of Radiology, Aarhus University Hospital, Aarhus, Denmark; 3 Institut des Vaisseaux et du Sang, Paris, France; 4 Department Cardiothoracic and Vascular Surgery, Aarhus University Hospital, Aarhus, Denmark; 5 Department Cardiology, Aarhus University Hospital, Aarhus, Denmark; 6 Department of Cardiology, Aarhus University Hospital, Brendstrupgaardsvej 100, DK-8200 Aarhus N, Denmark

## Abstract

**Background:**

Accelerated atherosclerosis is the main cause of late aortocoronary vein graft failure. We aimed to develop a large animal model for the study of pathogenesis and treatment of vein graft atherosclerosis.

**Methods:**

An autologous reversed jugular vein graft was inserted end-to-end into the transected common carotid artery of ten hypercholesteroemic minipigs. The vein grafts were investigated 12-14 weeks later with ultrasound and angiograpy in vivo and microscopy post mortem.

**Results:**

One minipig died during follow up (patent vein graft at autopsy), and one vein graft thrombosed early. In the remaining eight patent vein grafts, the mean (standard deviation) intima-media thickness was 712 μm (276 μm) versus 204 μm (74 μm) in the contralateral control internal jugular veins (P < .01). Advanced atherosclerotic plaques were found in three of four oversized vein grafts (diameter of graft > diameter of artery). No plaques were found in four non-oversized vein grafts (P < .05).

**Conclusions:**

Our model of jugular vein graft in the common carotid artery of hypercholesterolemic minipigs displayed the components of human vein graft disease, i.e. thrombosis, intimal hyperplasia, and atherosclerosis. Advanced atherosclerosis, the main cause of late failure of human aortocoronary vein grafts was only seen in oversized grafts. This finding suggests that oversized vein grafts may have detrimental effects on patient outcome.

## Background

Aortocoronary vein graft disease can be divided into three discrete, but pathophysiologically linked, processes: thrombosis, intimal hyperplasia, and atherosclerosis [1]. Vein graft failure is usually caused by thrombosis. In early vein graft failure, thrombosis is largely related to technical factors limiting graft blood flow. Vein grafts, that do not occlude early, develop intimal hyperplasia which rarely causes significant stenosis in itself. Intimal hyperplasia may, however, form the soil in which atherosclerotic plaques can develop. Late aortocoronary vein graft failure is most often caused by rupture of an atherosclerotic plaque in the graft leading to thrombotic occlusion [2]. Thereby, the pathogenesis of late graft failure parallels the pathogenesis of atherothrombosis in native coronary arteries [3]. The risk factors for atherosclerosis in aortocoronary vein grafts are also the same as for native coronary artery atherosclerosis with elevated plasma cholesterol being the most important risk factor [1], but vein graft atherosclerosis with atherothrombotic complications develops much more rapidly in aortocoronary vein grafts than in native coronary arteries [4].

Rupture of a lipid-rich atherosclerotic plaque with thrombosis leading to late graft failure remains a substantial clinical problem [1], so we aimed at developing a new animal model in which vein graft atherosclerosis, the primary cause of late vein graft failure, can be studied.

In adult hypercholesterolemic minipigs, we inserted an internal jugular vein graft segment into the common carotid artery (interposition graft). The vein grafts were examined with in vivo ultrasound and angiography and post mortem microscopic examination. The vein grafts displayed the entire spectrum of human aortocoronary vein graft disease, i.e. early thrombosis, intimal hyperplasia, and atherosclerosis. Atherosclerotic plaques were only seen in oversized vein grafts. This finding indicates that use of oversized vein grafts may have detrimental clinical effects through increased risk of atherosclerosis and late graft failure.

## Methods

This study was approved by the Danish Animal Experiments Inspectorate.

### Minipigs

Ten castrated male minipigs (9 months old) were used in this study. The minipigs are downsized pigs that are homozygous for the low density lipoprotein receptor mutation originally described in the Rapacz pigs [5]. The downsizing process, cholesterol levels, and coronary atherosclerosis have previously been reported and the applicability of this model has previously been reported [6-8].

### Feeding, bodyweight, and blood samples

**Figure 1:**
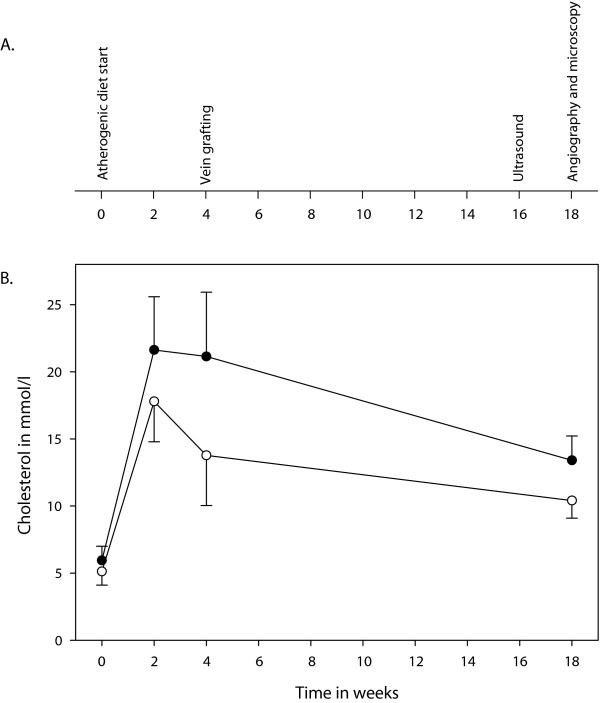
**Study design outline and total and low density lipoprotein cholesterol levels**. A. Study design outline. B. Total (filled circles) and low density lipoprotein (empty circles) cholesterol levels over the course of the study. Error bars represent standard deviations.

The study protocol is outlined in Figure 1A. During acclimatization, the minipigs were fed standard minipig diet and baseline blood samples were drawn. The minipigs were then fed an atherogenic diet for 4 weeks before the grafting procedure and for 14 weeks thereafter. Blood samples were drawn after 2, 4, 14, and 18 weeks on the atherogenic diet and analyzed for plasma total and low density lipoprotein cholesterol.

### Anestesia

For the surgical procedure and terminal angiography, the minipigs were preanesthetized with a mixture of azaperone (40 mg/ml, 0.1 ml/kg) and midazolam (5 mg/ml, 0.1 ml/kg). Venous access was obtained and the minipigs were sedated with propofol (10 mg/ml, 10-15 ml) followed by orothracheal intubation. The minipigs where then ventilated on a mechanical ventilator and anesthesia was maintained with sevoflurane and continuous infusion of fentanyl (50 μg/ml, 25-30 ml/min). In the first 3 days after the procedures, the minipigs received flunixin (50 mg/ml, 50 μl/kg/day).

For carotid ultrasound the minipigs were sedated with azaperone (40 mg/ml, 0.1 ml/kg) and midazolam (5 mg/ml, 0.1 ml/kg).

### Carotid vein graft procedure

The left common carotid was exposed, and an arterial sheath was inserted in the proximal direction. Upon removal of the sheath, the common carotid artery was transected, and a segment of the ipsilateral internal jugular vein was reversed and interpositioned into the common carotid artery with two end-to-end anastomoses. The vein graft segments were harvested immediately before engraftment. The segments were harvested without surrounding tissue and care was taken not to distend the veins during harvest and engraftment. The anastomoses were performed with continuous sutures. The left common carotid artery then consisted of an arterial segment proximal to the vein graft, a vein graft segment, and an arterial segment distal to the vein graft. The arterial sheath was inserted from the puncture site and in the proximal direction; this caused endothelial injury in the proximal arterial segment in relation to the procedure whereas the distal arterial segment was uninjured. To reduce the risk of local clotting and thrombosis, the minipigs received unfractioned heparin in relation to the procedures and aspirin (150 mg daily) thereafter.

### Carotid ultrasound and angiography

Carotid ultrasound including color Doppler and spectral Doppler investigation was performed (GE Logiq 9) 12 weeks after the surgery. A 4 MHz multi frequency curved array and a 10 MHz multifrequency linear transducer were used.

Angiography was performed immediately before termination of the study. Luminal diameters of the proximal arterial and vein graft segments were measured and angiographic stenosis degree (percent diameter stenosis) in the vein graft segment was calculated ([1-vein graft diameter/proximal arterial segment diameter]·100). A vein graft segment with an internal diameter exceeding the diameter of the carotid artery was considered oversized, whereas vein graft segments with an internal diameter equal to or smaller than the diameter of the carotid artery were not.

### Microscopic examination

The anesthetized minipigs were killed with an overdose of pentobarbital. The carotid arteries with vein graft segments were excised, pressure fixed with 4% formaldehyde at 100 mmHg for 1 hour, and immersion fixed for 6-12 hours. The arteries and vein grafts were sectioned at 4 mm intervals, paraffin embedded, and sectioned for microscopic examination. Hematoxylin and eosin, and trichrome-elastin stains were produced on all sections. To support interpretations based on these staining methods picrosirius red (collagen) and von Kossa staining in addition to immunohistochemistry for smooth muscle cells (Anti-Smooth Muscle Actin, DAKO M0851), macrophages (Anti-Lysozyme 12, DAKO A0099 [9]), and endothelium (Biotinylated Griffonia Simplicifolia Lectin I, Vector Laboratories B-1105) were produced on selected sections.

Digital images from microscopic examination were obtained and measurements on thickness and area were performed in ImageJ (National Institutes of Health). In the proximal and distal arterial common carotid artery segments, the intimal areas were measured in all sections. In the section with the largest intimal area, the intimal lesion was classified as described in Table 1 and intimal thickness was measured where it was thickest. In the vein grafts, intima and media were evaluated together because the intima-media border is ill-defined since the internal elastic lamina is ill-defined. In the vein grafts, the section with the most advanced intima-media lesion according to Table 1 was used in the analyses. Intima-media thickness was measured where the vein graft wall was thickest.

**Table 1:** Lesions in vein graft and artery proximal and distal to the graft

Segment	Artery proximal to graft^a^	Vein graft^a^	Artery distal to graft
**Lesion type **^b^			
Normal intima or adaptive intimal thickening^c^	1 (12.5%)	5 (62.5%)	8 (89%)
Intimal xanthoma (fatty streak)^d^	7 (87.5%)	0	1 (11%)
Plaque without necrotic core^e^	0	1 (12.5%)	0
Plaque with necrotic core^f^	0	2 (25%)	0
**Lesion thickness**			
Intimal (vein graft wall) thickness in μm^g^	82 (47)^h^	712 (276)	26 (32)^h^

Lesion types are given as count (frequency) and lesion thickness as mean (standard deviation)

^a ^n = 8: thrombosed segments were not classified

^b ^The distribution of lesion types differ between the three segments (P < .01) and also between the two arterial segments (P < .01)

^c ^Lesion consisting of normal connective tissue containing smooth muscle cells. No lipid accumulation or macrophages

^d ^Lesion consisting of normal intima except foam cell accumulation near lumen

^e ^Lesion with extracellular accumulation of lipid and connective tissue with fibrosis with or without calcification. No lipid-rich necrotic core

^f ^Lesion containing a lipid-rich necrotic core

^g ^In the vein grafts, the intima-media border is ill-defined and intima and media were considered together in the vein grafts

^h ^Intimal thickness was significantly different between the arterial segments (P < .05)

The scheme used to classify atherosclerotic lesions focuses on the lipid-rich necrotic core which is the most dangerous component by destabilizing plaques leading to rupture and thrombosis [10].

### Statistical analysis

Categorical values are presented as count (frequency) and numerical values as mean (standard deviation). Comparisons of numerical values were performed with Kruskal-Wallis tests, and test for correlations were performed with Spearman's rank correlation test. Proportions were compared with two-sample proportion calculator (Stata/IC 10.1 for Windows, StataCorp LP, TX, USA). P < .05 was considered statistically significant.

## Results

One minipig died during follow-up in relation to blood sampling, so 9 minipigs were available for in vivo carotid flow measurements by ultrasound and for in vivo angiography and post mortem microscopic examination.

### Body weights and plasma cholesterol

The minipigs had a steady low incline in bodyweight during the study, and all but one minipig (84 kg) weighed between 30 kg and 50 kg at the end of the study. The plasma total cholesterol levels rose markedly in response to the atherogenic diet, and this rise was mainly due to a rise in low density lipoprotein cholesterol (Figure 1B).

### Carotid flow and angiography

One vein graft showed evidence of early failure, i.e. early thrombotic occlusion. In the remaining eight minipigs, the carotid blood flow was 192 ml/min (80 ml/min), the peak systolic flow velocity was 99 cm/s (21 cm/s), and the end diastolic flow velocity was 12 cm/s (8 cm/s). The pulsatility index was 3.0 (1.3), and the resistive index was 0.87 (0.10) [11].

**Figure 2:**
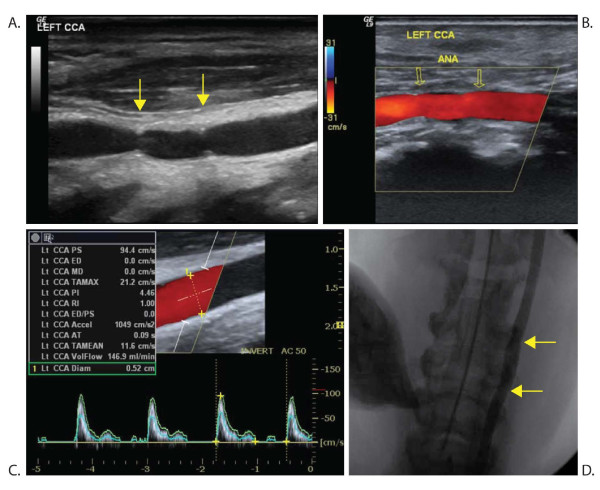
**Ultrasound and angiography of a common carotid artery with vein graft**. Gray-scale ultrasound (A) and angiography (D) showing the anatomy. Color Doppler (B) and spectral Doppler (C) investigation showing normal flow. Yellow arrows point at the anastomoses (ANA). CCA = common carotid artery. PI = pulsatility index. RI = resistive index.

Angiographically, the luminal diameter of the vein graft was 2% (7%) larger than that of the common carotid artery proximal to the graft (Figure 2) ranging from 13% below to 33% above the common carotid artery diameter.

### Microscopic examination

One vein graft had thrombosed early and was subsequently partially recanalized. The organized thrombus extended > 4 cm into the proximal arterial segment with previous endothelial injury but only < 1 cm into the distal arterial segment without endothelial injury. In the remaining 8 minipigs, there was no sign of thrombosis in the vein grafts.

**Figure 3:**
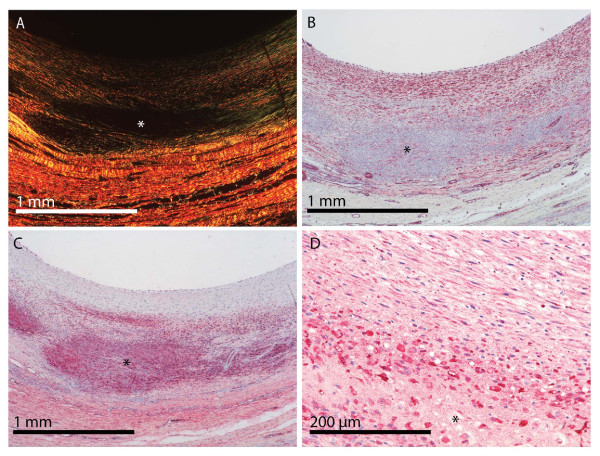
**Vein graft with atherosclerotic plaque containing a lipid-rich necrotic core**. A, Picrosirius red stain viewed under polarized light (collagen lights up), the necrotic core is defined by lack of collagen (black area marked with asterisk). B, immunohistochemical stain for smooth muscle cells demonstrates lack of smooth muscle cells in the necrotic core (asterisk) and plenty of smooth muscle cells in the fibrous cap separating the necrotic core from the lumen. C, lectin stain demonstrates neovascularization of the vein graft but also stains macrophages and erythrocyte membranes including the debris from these cell types that can be found in the necrotic core (asterisk). D, immunohistochemical stain for macrophages demonstrates numerous macrophages in the border of the necrotic core (asterisk).

Atherosclerotic plaque had developed in three minipigs. In one minipig, a plaque without a lipid-rich necrotic core was found in the intima-media layer and in two minipigs plaques with lipid-rich necrotic cores were found (Figure 3). In the proximal arterial segment that was subjected to endothelial injury at the time of engraftment, intimal xanthoma (fatty streak) was found in all but one minipig. In contrast, in the distal arterial segment that was not subjected to endothelial injury, normal intima or intimal thickening was found in all but one minipig (Table 1).

The intima-media thickness in the 8 patent vein grafts was 712 μm (276 μm). In contralateral control internal jugular veins (not perfusion fixed), the intima-media thickness was 204 μm (74 μm). The difference in intima-media thickness between normal jugular veins and the arterialized internal jugular vein grafts was statistically significant (P < .01). In the 5 vein grafts without plaque, the intima-media thickness was 608 μm (254 μm) and significantly larger than in control veins (P < .05). In the 3 vein grafts with plaque, the intima-media thickness was 882 μm (258 μm) and significantly larger than in control veins (P < .05). There was no significant difference between intima-media thickness in the vein grafts with or without plaque (P = .18).

### Relation between microscopic examination and cholesterol levels, flow, and angiography

Wall thickness did not correlate with cholesterol levels, flow, or angiographic stenosis. There was no difference in cholesterol levels between pigs with or without vein graft plaque. Neither was there difference in flow between vein grafts with or without plaque.

The atherosclerotic plaques were found in 3 of 4 vein grafts with a larger luminal diameter than the common carotid artery, whereas vein grafts with a luminal diameter less than the common carotid artery diameter never contained plaque. This represents a statistically significant difference in plaque occurrence between the veins with luminal diameters larger or smaller than the common carotid artery diameter (P < .05).

## Discussion

We present a large animal model of human-like vein graft disease, including advanced atherosclerosis, in hypercholesterolemic minipigs. Our findings suggest that oversized vein grafts promote the development of atherosclerosis in the graft. Since thrombosis elicited by vein graft atherosclerosis is the main cause of late aortocoronary vein graft failure, this finding may have important clinical implications.

### Hypercholesterolemia and vein graft disease

Early aortocoronary vein graft failure with thrombotic occlusion is not related to hypercholesterolemia but rather to surgery related technical factors [1]. Aortocoronary vein graft intimal hyperplasia is ubiquitous in grafts that do not fail early and is regarded as an adaptive response that does not depend on elevated plasma cholesterol levels [1,12]. In contrast, aortocoronary vein graft atherosclerosis and late graft failure is more common in patients with higher plasma cholesterol levels [13,14], and plasma cholesterol lowering decreases the risk of late aortocoronary vein graft failure in patients [15,16].

This is in agreement with findings in mice and rabbits, where normocholesterolemic animals consistently develop vein graft intimal hyperplasia but not vein graft atherosclerosis which has only been observed in severely hypercholesterolemic animals [17,18]. In accordance, normocholesterolemic pigs consistently develop vein graft intimal hyperplasia but not vein graft atherosclerosis [19]. Neither did pigs with modestly elevated cholesterol levels [20]. Our report is the first of vein graft atherosclerosis with advanced plaques containing the dangerous lipid-rich necrotic core in pigs [21].

### Flow and vein graft disease

In humans, decreased risk of late aortocoranary vein graft failure was found in grafts bypassing severely stenotic lesions (stenosis degree > 70%) versus grafts bypassing less stenotic lesions (stenosis degree < 70%) [22]. Additionally, aneurismal aortocoronary vein grafts have a higher risk of late failure in humans [23]. Based on such findings, it has been hypothesized that lower graft flow through lower endothelial shear stress in the vein graft increases the risk of atherothrombosis and late graft failure.

In normocholesterolemic rabbits and dogs, low flow influences cytokine levels in a direction suspected to be proatherogenic [24], and leads to increased intimal hyperplasia [25]. But a relation between decreased flow and increased vein graft atherosclerosis has not been shown previously in hypercholesterolemic animals. We report a significant relation between vein graft diameter and atherosclerosis in pigs. This finding points to a proatherogenic effect of low endothelial shear stress in vein grafts and may indicate that oversized vein grafts carry increased risk of atherosclerosis and consequential late failure.

In mice, typically the caval vein or external jugular is interpositioned into the carotid artery. In rabbits, usually the external jugular vein is interpositioned into the carotid artery. These vein grafts all have a larger diameter than the native carotid artery which may augment atherosclerosis development in these models and conceal the atheroprotective effect of vein graft diameter below grafted artery diameter. In most pig models, anastomoses are created with the vein and artery ends beveled obliquely to 45 degrees. In this study, the ends were not beveled.

### Limitations of this study

We did not assess endothelial shear stress directly, but since the grafts with plaque have larger diameters for the same flow we infer a pathogenic effect mediated through shear stress.

The plaques developed in animal models may not be entirely representative of human disease despite bearing large resemblance, and we did not observe plaque rupture and thrombosis leading to late graft failure. Reproducible animal models of spontaneous plaque rupture and thrombosis do not exist. Although the process may occur rarely, it may occur silently and be missed. With extension of the study duration plaque rupture could occur, but an extension a study like this is costly due to costs of stable facilities and atherogenic diets. Taking our findings into consideration, deliberately using oversized vein grafts would lead to a higher frequency atherosclerotic lesions and thereby increase the likelihood of observing plaque rupture.

As in many other animal studies, we used grafts interpositioned with two end-to-end anastomoses although this differs from the usual use of end-to-side and side-to-side anastomoses used in human coronary artery bypass surgery.

## Conclusions

We present a human-like model of vein graft disease including advanced atherosclerosis in hypercholesterolemic minipigs. This is the first report of advanced vein graft atherosclerosis in pigs. We found that oversized vein grafts (diameter of graft > diameter of artery) increased the risk of advanced vein graft atherosclerosis. Since thrombosis elicited by vein graft atherosclerosis is the main cause of late vein graft failure, this finding indicates that oversized vein grafts may have detrimental effects on clinical outcome.

## Competing interests

The authors declare that they have no competing interests.

## Authors' contributions

TT: conception and design, surgical procedures, angiography, microscopy, data analysis, manuscript draft. MKH: conception and design, surgical procedures, data analysis, manuscript revision. AH: conception and design, ultrasound, data analysis, manuscript revision. LD: conception and design, data analysis, manuscript revision. WPP: conception and design, surgical procedures, data analysis, manuscript revision. HEB: conception and design, data analysis, manuscript revision. EF: conception and design, microscopy, data analysis, manuscript revision. All authors read and approved the final manuscript.

## Pre-publication history

The pre-publication history for this paper can be accessed here:

http://www.biomedcentral.com/1471-2261/12/24/prepub

## References

[B1] Motwani JG Topol EJ Aortocoronary saphenous vein graft disease: pathogenesis, predisposition, and prevention Circulation 1998 97 916 931 9521341 10.1161/01.cir.97.9.916

[B2] Qiao JH Walts AE Fishbein MC The severity of atherosclerosis at sites of plaque rupture with occlusive thrombosis in saphenous vein coronary artery bypass grafts Am Heart J 1991 122 955 958 10.1016/0002-8703(91)90457-S 1927881

[B3] Thim T Hagensen MK Bentzon JF Falk E From vulnerable plaque to atherothrombosis J Intern Med 2008 263 506 516 10.1111/j.1365-2796.2008.01947.x 18410594

[B4] Falk E Thuesen L Pathology of coronary microembolisation and no reflow Heart 2003 89 983 985 10.1136/heart.89.9.983 12923001 PMC1767861

[B5] Hasler-Rapacz J Ellegren H Fridolfsson AK Kirkpatrick B Kirk S Andersson L Rapacz J Identification of a mutation in the low density lipoprotein receptor gene associated with recessive familial hypercholesterolemia in swine Am J Med Genet 1998 76 379 386 10.1002/(SICI)1096-8628(19980413)76:5<379::AID-AJMG3>3.0.CO;2-I 9556295

[B6] Thim T Human-like atherosclerosis in minipigs: a new model for detection and treatment of vulnerable plaques Dan Med Bull 2010 57 B4161 20591344

[B7] Thim T Hagensen MK Drouet L Bal dit SC Bonneau M Granada JF Nielsen LB Paaske WP Botker HE Falk E Familial hypercholesterolaemic downsized pig with human-like coronary atherosclerosis: a model for preclinical studies EuroIntervention 2010 6 261 268 10.4244/EIJV6I2A42 20562079

[B8] Thim T Hagensen MK Wallace-Bradley D Granada JF Kaluza GL Drouet L Paaske WP Botker HE Falk E Unreliable assessment of necrotic core by virtual histology intravascular ultrasound in porcine coronary artery disease Circ Cardiovasc Imaging 2010 3 384 391 10.1161/CIRCIMAGING.109.919357 20460496

[B9] Falk E Fallon JT Mailhac A Fernandez-Ortiz A Meyer BJ Weng D Shah PK Badimon JJ Fuster V Muramidase: A useful monocyte/macrophage immunocytochemical marker in swine, of special interest in experimental cardiovascular disease Cardiovasc Pathol 1994 3 183 189 10.1016/1054-8807(94)90028-0 25990995

[B10] Virmani R Kolodgie FD Burke AP Farb A Schwartz SM Lessons from sudden coronary death: a comprehensive morphological classification scheme for atherosclerotic lesions Arterioscler Thromb Vasc Biol 2000 20 1262 1275 10.1161/01.ATV.20.5.1262 10807742

[B11] Nicolaides AN Shifrin EG Bradbury A Dhanjil S Griffin M Belcaro G Williams M Angiographic and duplex grading of internal carotid stenosis: can we overcome the confusion? J Endovasc Surg 1996 3 158 165 10.1583/1074-6218(1996)003<0158:AADGIC>2.0.CO;2 8798134

[B12] Owens CD Adaptive changes in autogenous vein grafts for arterial reconstruction: Clinical implications J Vasc Surg 2010 51 736 746 10.1016/j.jvs.2009.07.102 19837532 PMC2834835

[B13] Campeau L Enjalbert M Lesperance J Bourassa MG Kwiterovich P Jr Sniderman A Wacholder S The relation of risk factors to the development of atherosclerosis in saphenous-vein bypass grafts and the progression of disease in the native circulation. A study 10 years after aortocoronary bypass surgery N Engl J Med 1984 311 1329 1332 10.1056/NEJM198411223112101 6333635

[B14] Lie JT Lawrie GM Morris GC Jr Aortocoronary bypass saphenous vein graft atherosclerosis. Anatomic study of 99 vein grafts from normal and hyperlipoproteinemic patients up to 75 months postoperatively Am J Cardiol 1977 40 906 914 10.1016/0002-9149(77)90041-8 303864

[B15] Campeau L Hunninghake DB Knatterud GL White CW Domanski M Forman SA Forrester JS Geller NL Gobel FL Herd JA Aggressive cholesterol lowering delays saphenous vein graft atherosclerosis in women, the elderly, and patients with associated risk factors. NHLBI post coronary artery bypass graft clinical trial. Post CABG Trial Investigators Circulation 1999 99 3241 3247 10385497 10.1161/01.cir.99.25.3241

[B16] Cashin-Hemphill L Mack WJ Pogoda JM Sanmarco ME Azen SP Blankenhorn DH Beneficial effects of colestipol-niacin on coronary atherosclerosis. A 4-year follow-up JAMA 1990 19 264 3013 3017 2243429

[B17] Zou Y Dietrich H Hu Y Metzler B Wick G Xu Q Mouse model of venous bypass graft arteriosclerosis Am J Pathol 1998 153 1301 1310 10.1016/S0002-9440(10)65675-1 9777962 PMC1853044

[B18] Zwolak RM Kirkman TR Clowes AW Atherosclerosis in rabbit vein grafts Arteriosclerosis 1989 9 374 379 10.1161/01.ATV.9.3.374 2719597

[B19] Angelini GD Bryan AJ Williams HM Soyombo AA Williams A Tovey J Newby AC Time-course of medial and intimal thickening in pig venous arterial grafts: relationship to endothelial injury and cholesterol accumulation J Thorac Cardiovasc Surg 1992 103 1093 1103 1597973

[B20] Angelini GD Lloyd C Bush R Johnson J Newby AC An external, oversized, porous polyester stent reduces vein graft neointima formation, cholesterol concentration, and vascular cell adhesion molecule 1 expression in cholesterol-fed pigs J Thorac Cardiovasc Surg 2002 124 950 956 10.1067/mtc.2002.127004 12407378

[B21] Schachner T Laufer G Bonatti J In vivo (animal) models of vein graft disease Eur J Cardiothorac Surg 2006 30 451 463 10.1016/j.ejcts.2006.06.015 16870461

[B22] Roth JA Cukingnan RA Brown BG Gocka E Carey JS Factors influencing patency of saphenous vein grafts Ann Thorac Surg 1979 28 176 183 10.1016/S0003-4975(10)63777-0 314277

[B23] Solymoss BC Nadeau P Millette D Campeau L Late thrombosis of saphenous vein coronary bypass grafts related to risk factors Circulation 1988 78 I140 I143 3261650

[B24] Onohara T Okadome K Yamamura S Komori K Ishii T Odashiro T Sugimachi K Impaired endothelial prostacyclin production of the canine vein graft in a poor distal runoff limb Surgery 1993 113 700 708 8506529

[B25] Morinaga K Okadome K Kuroki M Miyazaki T Muto Y Inokuchi K Effect of wall shear stress on intimal thickening of arterially transplanted autogenous veins in dogs J Vasc Surg 1985 2 430 433 3999234

